# Improving the success rate of human corneal endothelial cell cultures from single donor corneas with stabilization medium

**DOI:** 10.1007/s10561-017-9665-y

**Published:** 2017-10-17

**Authors:** D. Spinozzi, A. Miron, M. Bruinsma, J. T. Lie, I. Dapena, S. Oellerich, G. R. J. Melles

**Affiliations:** 1 Netherlands Institute for Innovative Ocular Surgery, Laan op Zuid 88, 3071AA Rotterdam, The Netherlands; 2 Melles Cornea Clinic Rotterdam, Rotterdam, The Netherlands; 3 Amnitrans EyeBank Rotterdam, Rotterdam, The Netherlands

**Keywords:** Human corneal endothelial cells, Stabilization medium, Cell culture, Cell isolation, Cell morphology, Cell viability

## Abstract

Main objective of this study was to improve the success rate of human corneal endothelial cell (hCEC) cultures from single donor corneas. We could show that the use of stabilization medium *prior* to cell isolation may have a positive effect on the success rate of hCEC cultures from single research-grade donor corneas by allowing growth of otherwise possibly not successful cultures and by improving their proliferative rate. hCEC were obtained from corneo-scleral rims of 7 discarded human research-grade cornea pairs. The Descemet membrane–endothelium (DM–EC) sheets of each pair were assigned to 2 experimental conditions: (1) immediate cell isolation after peeling, and (2) storage of the DM–EC sheet in a growth factor-depleted culture medium (i.e. stabilization medium) for up to 6 days *prior* to cell isolation. hCEC isolated by enzymatic digestion were then induced to proliferate on pre-coated culture plates. The success rate of primary cultures established from single donor corneas were higher for DM–EC sheets kept in stabilization medium before cell isolation. All cultures (7/7) initiated from stabilized DM–EC sheets were able to proliferate up to the third passage, while only 4 out of 7 cultures initiated from freshly peeled DM–EC sheets reached the third passage. In addition, for the 4 successful paired cultures we observed a faster growth rate if the DM–EC sheet was pre-stabilized prior to cell isolation (13.8 ± 1.8 vs 18.5 ± 1.5 days, *P* < 0.05). Expression of the phenotypical markers Na^+^/K^+^-ATPase and ZO-1 could be shown for the stabilized cultures that successfully proliferated up to the third passage.

## Introduction

Human corneal endothelial cells (hCEC) are crucial for maintaining corneal transparency, since loss of their functionality owing to endothelial diseases or trauma, results in corneal swelling and loss of corneal clarity (Gagnon et al. 1997; Joyce 2003). Because of the limited proliferation capacity of hCEC in vivo (Schultz et al. 1984), replacement of diseased or damaged endothelium by healthy donor cells by means of corneal transplantation, is currently the only effective treatment option to restore patients’ vision (Engelmann et al. 2004). Over the last decade, corneal transplantation for treating endothelial disease, has swiftly advanced from full-thickness penetrating keratoplasty, to the more selective endothelial keratoplasty techniques. Descemet membrane endothelial keratoplasty (DMEK) is the most selective of these techniques to date and specifically replaces the recipient’s diseased endothelium and Descemet membrane (DM) by a healthy donor DM–endothelium (DM–EC) sheet (Melles and Dapena 2014; Melles et al. 2006). Although this method has several advantages over traditional penetrating keratoplasty (shortens the recovery time, reduces the risk of inflammation and graft rejection), one of its limitations is the shortage of high quality healthy donor tissue. This has led to considerable interest in the development of new strategies to increase the pool of available donor tissue, such as the introduction of hemi- and quarter-DMEK (Gerber-Hollbach et al. 2016; Muller et al. 2017), in which the donor DM–EC is divided in 2 and 4 pieces, respectively, allowing a much more efficient use of donor tissue.

Transplantation of in vitro expanded cultured hCEC from healthy donor corneas, would be an alternative approach that could possibly solve donor tissue shortage (Choi et al. 2010; Koizumi et al. 2012). In most in vitro culture protocols, hCEC isolated from several donor corneas are pooled together and induced to proliferate (Nakahara et al. 2013; Okumura et al. 2015). However, results have been variable and often with limited success (Peh et al. 2011a, b). In addition, this approach might not be suitable for future clinical application, because of lack of donor traceability and increased antigen load that would significantly increase the risk of allograft rejection. Therefore, we aimed to culture hCEC from single donor corneas. However, one of the main challenges here is the establishment of a reproducible protocol for the in vitro propagation, since the proliferative capacity of hCEC is influenced by many factors including cell density, and this may be lower than required when isolating hCEC from one single donor cornea. A low hCEC density at initiation of culture may induce endothelial-to-mesenchymal transition and might have a general negative impact on morphology and proliferation in vitro (Peh et al. 2013).

In a recent report by Peh et al., a dual media culture approach before passaging cultured hCEC was described, in which a serum-supplemented medium was shown to prevent endothelial-to-mesenchymal transition of hCEC expanded in proliferative medium and to conserve hCEC morphology in vitro (Peh et al. 2015). Based on this, we hypothesized that preserving the entire DM–EC sheet prior to hCEC isolation in a similar serum-supplemented medium with no added growth factors (stabilization medium) would be beneficial for culturing hCEC from single donor corneas (i.e. without pooling several donor corneas to establish a culture). To minimize the effect of donor variation, we chose a paired donor cornea approach in which hCEC of one cornea of each pair were immediately isolated after peeling the DM–EC sheet, whereas the DM–EC sheet of the contralateral cornea was kept in stabilization medium for 4–6 days prior to hCEC isolation. The success of establishing stable hCEC cultures as well as their growth rates were assessed.

## Materials and methods

### Materials

Dulbecco’s modified eagle medium (DMEM), fetal bovine serum (FBS), Dulbecco’s phosphate-buffered saline (PBS), TrypLE™ Express (TE), ascorbic acid 2-phosphate, collagenase from *Clostridium histolyticum* (Type A), paraformaldehyde (PFA), bovine serum albumin (BSA), 4′,6-diamidino-2-phenylindole (DAPI), and Triton X-100 were purchased from Sigma-Aldrich Chemistry BV (Zwijndrecht, The Netherlands). Pen/Strep Pre-Mix was purchased from Carl Roth GmbH + Co. KG (Karlsruhe, Germany). Fibronectin, collagen, and albumin (FNC) coating mix was purchased from Athena ES™ (Baltimore, MD, USA). Antibodies were obtained from Life Technology Europe BV (Bleiswijk, The Netherlands). Trypan Blue solution 0.04% (Hippocratech, Rotterdam, The Netherlands) was used to assess the vitality of hCEC during the isolation and culture protocol as well as to ensure the visibility of the DM–EC sheet during preparation.

### Research-grade human corneoscleral tissues

Seven discarded research-grade human cornea pairs from 2 female and 5 male donors with a mean age of 69 (± 15) years (range 42–80 years, Table 1) were included in the study. All donor corneas were obtained from Amnitrans EyeBank Rotterdam and had an intact and viable endothelium, but were unsuitable for transplantation. In all cases, the donors had stated to have no objection against transplant-related research.

**Table 1:** Demographics of donor data

Donor information	Indicators
*Donor data*	
Gender	
Female	2
Male	5
Mean age (± SD), yrs (range)	69 (± 15), (42–80)
Mean storage time (± SD), days (range)	16 (± 5), (9–23)
Cause of death	
Cardio/stroke	2
Infectious	1
Respiratory	1
Cancer	1
Other	2

Mean storage time = time between death and culture of first isolated DM–EC tissue; SD = standard deviation; yrs = years

### Donor tissue protocol

Primary hCEC were isolated from DM–EC sheets using a two-step, peel-and-digest method. The protocol for harvesting the DM–EC sheets has been described previously (Groeneveld-van Beek et al. 2013; Lie et al. 2008). Briefly, after decontamination of the globes, corneo-scleral rims were excised within 36 h post-mortem and stored in preservation medium (CorneaMax, Eurobio, Courtaboeuf, France) at 31 °C until further processing. To peel the DM–EC sheet, corneo-scleral rims were placed endothelial-side-up on a custom made holder with a suction cup. DM–EC was then stained with 0.04% Trypan Blue solution for 10 s to visualize Schwalbe’s line. DM–EC including trabecular meshwork was loosened over 360°. By holding the trabecular meshwork with fine forceps and making gentle centripetal movements, the DM–EC sheet was carefully peeled from the posterior stroma. After removing the trabecular meshwork, a ‘Descemet-roll’ formed spontaneously with the endothelium laying on the outer side. The DM–EC sheets obtained from each pair as described above were processed further by (1) immediate isolation of hCEC from the DM–EC sheet (non-stabilized hCEC), and (2) by storing the entire contralateral DM–EC sheet in stabilization medium (SM, Table 2) (stabilized-hCEC) first for 4–6 days before hCEC isolation.

**Table 2:** Supplemented media used in the culture of human corneal endothelial cells

Basal medium	Serum (%)	Growth factors and supplements
[PM] DMEM (Shima et al. 2011)	15	2 mM l-glutamine 2 ng/ml bFGF 0.3 mM l-ascorbic acid 2-phosphate 10,000 U-ml pen/strep
[SM] DMEM (Peh et al. 2015)	15	10,000 U-ml pen/strep

*PM* proliferation medium, *SM* stabilization medium

### Isolation and growth of human corneal endothelial cells

**Fig. 1:**
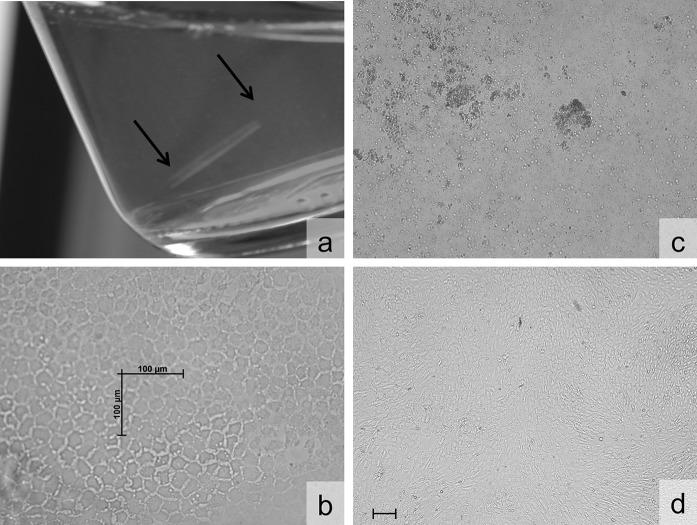
Macroscopic and light microscopic images of the endothelium after DM–EC sheet isolation from a discarded donor corneo-scleral rim. **a** The DM–EC sheet in culture medium. **b** After stripping of the DM–EC sheet, no marked changes in endothelial cells occur throughout the DM–EC sheet. **c** DM–EC sheet after 4 h of digestion in Collagenase A diluted in DMEM. **d** Confluent hCEC culture at P0 (scale bars = 100 µm)

For both conditions (non-stabilized and stabilized DM–EC sheets), hCEC were isolated from the DM–EC sheets by exposing them to a 2 mg/ml collagenase A (in DMEM) solution for 3–6 h at 37 °C and 5% CO_2_ to dislodge hCEC from DM, which resulted in tightly packed hCEC clusters (Fig. 1). hCEC clusters were further dissociated into single cells with TrypLE™ for 5 min at 37 °C and the resulting cell suspension was centrifuged at 500 rpm for 5 min at 37 °C. The cell pellet was re-suspended in Proliferation Medium (PM, Table 2) and plated onto culture well plates previously coated with FNC coating mix. From each culture, 10 µl of cell suspension was collected to perform an automatic cell counting using a Spark™ 10 M multimode microplate reader (Tecan Trading AG, Männedorf, Switzerland). The cultures were kept in a humidified atmosphere at 37 °C and 5% CO_2_. For routine maintenance, every 2–3 days medium was replaced with fresh proliferation medium. When primary cultures of hCEC reached the stationary phase with 80–90% confluence (approximately after 3 weeks) (Fig. 1), proliferation medium was replaced with stabilization medium for the next 2–4 days before passaging to enhance the morphology of the expanded hCEC (Ishino et al. 2004; Peh et al. 2015). Upon passaging, cultured hCEC were treated with 0.05% Trypsin/0.02% EDTA solution (TE) for 15 min at 37 °C and 5% CO_2_, the cell pellet was re-suspended in proliferation medium, and cells were sub-cultured at a 1:2 splitting ratio on FNC-coated culture well plates. The morphology of the cultured hCEC at confluence and during expansion was observed with an AxioVert.A1 microscope with AxioCam ERc 5 s stand-alone functionality camera (Zeiss, Oberkochen, Germany).

### Immunofluorescence

ZO-1 and Na^+^/K^+^-ATPase are phenotypical markers for hCEC. To visualize ZO-1 and Na^+^/K^+^-ATPase, hCEC were cultured either on glass coverslips or directly on FNC-coated well plates and fixed in 4% paraformaldehyde for 15 min at room temperature. Following fixation, hCEC were first washed with PBS and then permeabilized using permeabilization buffer (0.1% Triton X-100 in PBS) and then rinsed with blocking buffer (5% bovine serum albumin (BSA) in PBS) for 1 h to prevent non-specific staining. Blocking buffer was also used for primary and secondary antibody dilutions. Incubation with primary antibodies (anti-ZO-1/TJP1 at 1:100, and anti-Na^+^/K^+^-ATPase at 1:100) was done overnight at 4 °C, followed by a secondary antibody incubation (1:200) for 1 h at room temperature. After washing with PBS, the samples were stained with DAPI to visualize the nuclear DNA, and then imaged using an inverted fluorescence microscope connected to a camera (Axiovert, Zeiss).

### Statistical analysis

A student *t* test was performed for outcome comparison between stabilized and non-stabilized DM–EC sheets (SPSS for Windows software, version 15.0, SPSS, Inc.). *P* values of less than 0.05 were considered statistically significant.

## Results

Endothelial cell density (ECD) determined in the eye bank before DM–EC sheet harvesting was on average 2536 ± 766 cells/mm^2^, with no significant difference between the two groups (*P* = 0.10). All other donor-related parameters were identical for both groups due to the paired-donor approach. Cell concentration in both groups (cells/ml) was determined prior to seeding the cells onto the FNC-coated well plates. Average cell concentration was higher for non-stabilized hCEC (7197 ± 5860 cells/ml) than for stabilized hCEC (2435 ± 1656 cells/ml) (*P* > 0.05).

**Fig. 2:**
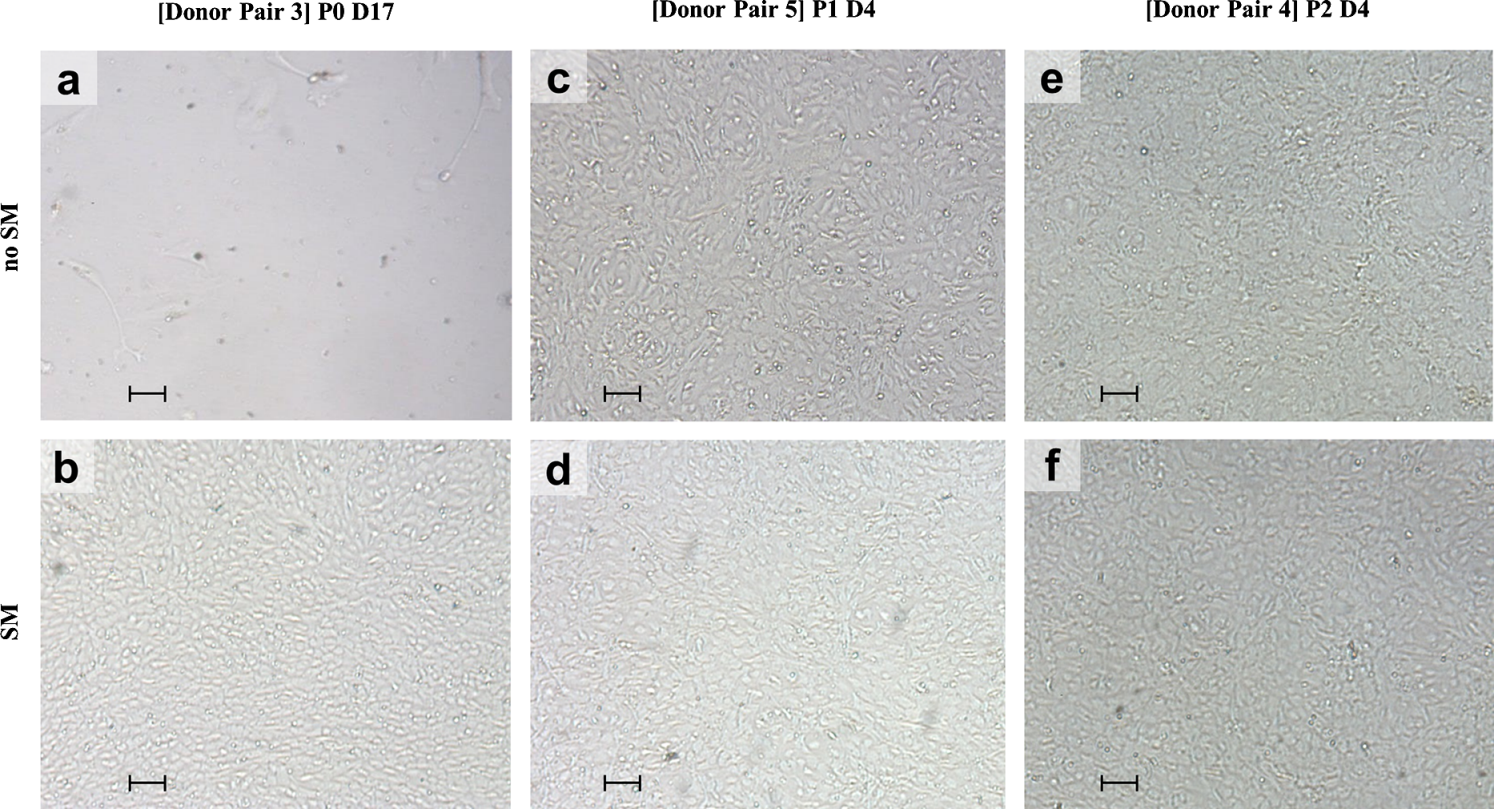
Morphology of cultured hCEC from P0 to P2. Photographs representing the morphology of hCEC isolated from non-stabilized (no SM) and stabilized (SM) DM–EC sheets. Light microscopy images of cultured hCEC are shown for three corneas pairs at initiation of culture (P0), passage 1 (P1), and passage 2 (P2). **a**, **b** Donor pair 3: P0 at day 17 (D17) at the end of the proliferative phase, before first passaging. **c**, **d** Donor pair 5: P1 at day 4 (D4), before second passaging. **e**, **f** Donor pair 4: P2 at day 4 (D4) before third passaging (scale bars = 100 µm)

**Fig. 3:**
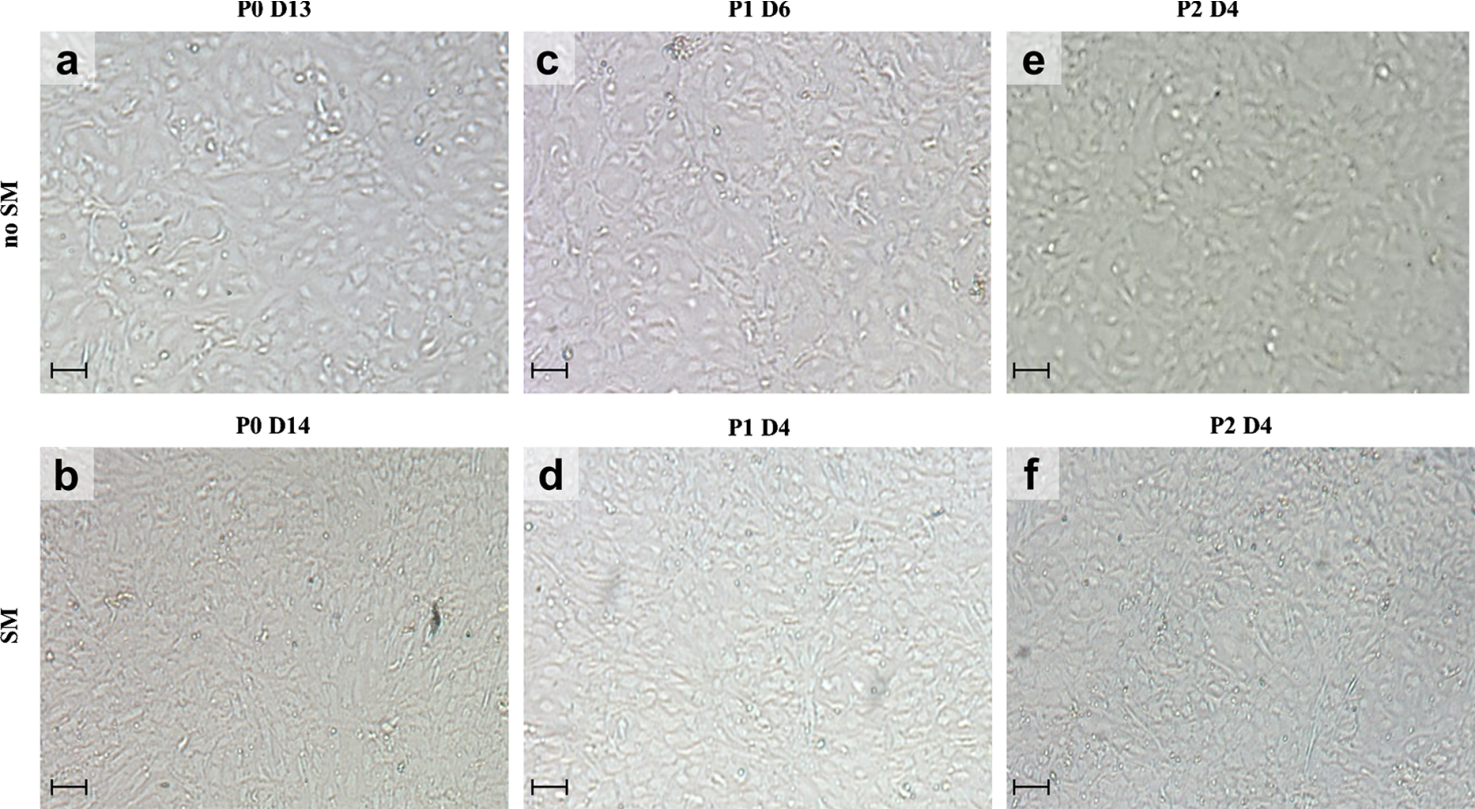
Light microscopy images of successful paired hCEC cultures from P0 to P2. Confluent hCEC cultures isolated from both non stabilized (no SM) and stabilized (SM) DM–EC sheets of donor pair 4. Cell density and morphology were evaluated by light microscopy at P0 (**a**, **b**), P1 (**c**, **d**), and P2 (**e**, **f**) (scale bars = 100 µm)

While all cell cultures (7/7) established from stabilized hCEC could be expanded up to the third passage, only 5/7 cultures of the non-stabilized hCEC reached P1 of which 4 could reach P2 (Table 3; Figs. 2, 3). In these cultures we observed a faster growth rate (time to reach confluence during P0) for stabilized hCEC (13.8 ± 1.8 days) compared to the non-stabilized hCEC (18.5 ± 1.5 days, *P* < 0.05) (Table 3) while the characteristic endothelial cobblestone morphology was maintained (Fig. 2). Independent of pre-stabilization or not, after the first passage was successful, cell morphology and growth rate were similar between the groups (Table 3, donor pairs 2, 4, 5, and 6) (Fig. 2e, f). An example of hCEC from one donor pair where the culture was successful independent of prior stabilization is shown in Fig. 3 (Table 3, Culture 5).

**Table 3:** Number of days per passage and culture prior to confluence

Passage no.	Donor pair 1 [80 yrs]		Donor pair 2 [53 yrs]		Donor pair 3 [79 yrs]		Donor pair 4 [42 yrs]		Donor pair 5 [72 yrs]		Donor pair 6 [76 yrs]		Donor pair 7 [79 yrs]	
	Non-SM	SM	Non-SM	SM	Non-SM	SM	Non-SM	SM	Non-SM	SM	Non-SM	SM	Non-SM	SM
P0	10	13	20	16	–	23	17	14	17	14	20	11	–	14
P1	–	6	6	4	–	6	6	6	6	4	17	4	–	4
P2	–	6	9	7	–	7	4	4	4	4	4	7	–	2

*SM* stabilization medium prior to cell isolation, *non-SM* no stabilization medium prior to cell isolation, *yrs* years

For the 2/7 non-stabilized hCEC that could not complete P0 (Table 3, donor pairs 3 and 7), an abnormal morphology (with elongated fibroblast-like shape) was observed and hCEC were unable to establish confluence. One non-stabilized hCEC culture successfully reached confluence in P0 but not in P1 (Table 3, donor pair 1). Here, cells became gradually stretched and also did not obtain confluence.

**Fig. 4:**
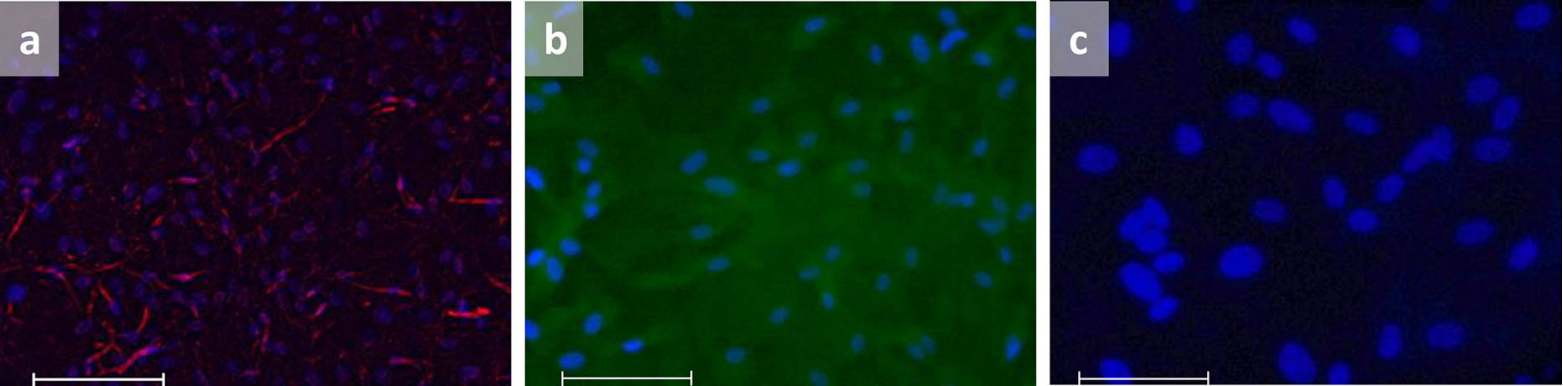
Characterization and expression of cultured hCEC. Illustrative series of fluorescent images showing the expression of Na^+^/K^+^-ATPase and ZO-1 of hCEC after passage 2 by immunocytochemistry. **a** Immunostaining of Na^+^/K^+^-ATPase. **b** Immunostaining of ZO-1. **c** Isotype matched IgG_1_ negative control. DAPI was used in all experiments for nuclei staining (scale bars = 100 µm)

Expression of the phenotypical markers Na^+^/K^+^-ATPase and ZO-1 could be shown for the stabilized hCEC that successfully reached P2 (Fig. 4). Na^+^/K^+^-ATPase expression had a more diffuse pattern all over the cell surfaces, whereas ZO-1 was mostly expressed on the cell borders.

## Discussion

To this day, an impressive amount of protocols for isolation and proliferation of hCEC have been proposed, with a main focus on media composition and use of various specific growth factors (Hoppenreijs et al. 1994; Shima et al. 2011). However, isolation and proliferation of hCEC in vitro from human donors remains challenging, especially when cultures have to be established from hCEC which derive from just one donor cornea and the baseline parameters of this research-grade cornea are not optimal. We hypothesized that the success rate of establishing cell cultures of hCEC isolated from single research-grade corneas could be improved by storing freshly peeled DM–EC sheets for 4–6 days in a stabilization medium prior to cell isolation. In this study, we found that 100% of the hCEC cultures initiated from stabilized DM–EC sheets propagated well over two passages whereas cultivated hCEC isolated from the contralateral cornea and expanded immediately after DM–EC sheet harvesting had a success rate of 57%.

We observed that the initial cell concentration before seeding was higher for hCEC isolated from non-stabilized DM–EC sheets than for hCEC isolated from stabilized DM–EC sheets. The most likely explanation for this result is that our stabilization medium affects the presence of viable and non-viable cells. It is known that both the length of organ culture storage time of the corneo-scleral rim (Albon et al. 2000; Armitage and Easty 1997; Borderie et al. 1998; Pels and Schuchard 1983; Gauthier et al. 2016), and mechanical stress caused by peeling the DM–EC sheet may increase the number of non-viable hCEC (Bhogal et al. 2016; Marty et al. 2016). This may imply that the initially higher cell concentration before seeding for the non-stabilized hCEC, i.e. isolated immediately after peeling, measured a population of both viable and non-viable cells. The lower cell concentration for the stabilized hCEC may then be explained by a ‘loss’ of non-viable cells during the stabilization period with mainly viable cells remaining on the DM–EC sheet. Thus, we may find a higher concentration of hCEC after isolating them from non-stabilized DM–EC sheets compared to stabilized DM–EC sheets because of the presence of non-viable cells in the former which are lacking in the latter.

It is known that non-viable cells may negatively impact the cells in their immediate vicinity (Gregory et al. 2009). Since in vitro cell cultures have no mechanism to remove non-viable cells, in the non-stabilized cultures the behavior of viable hCEC may therefore have been negatively influenced by their non-viable neighbors. This also suggests that by storing the DM–EC sheet for some days in stabilization medium, we were able to isolate and culture a more viable hCEC population. Therefore, it is of the utmost importance to remove the apoptotic cells before seeding the cells, as they produce various factors that may negatively impact their viable neighbors (Gregory and Pound 2010). This may explain the improved growth characteristics of stabilized hCEC compared to non-stabilized hCEC. In three pairs where we could not establish a culture from non-stabilized hCEC, we were able to culture stabilized hCEC from the contralateral cornea over several passages with normal morphology and expression of markers characteristic of human corneal endothelium: Na^+^/K^+^-ATP and ZO-1.

It is well known that the conditions that may lead to a successful hCEC culture are quite precarious; i.e. an extended storage time before isolation and culture, a high donor age (Joyce 2003, 2012; Joyce and Zhu 2004), and a low yield of hCEC at the start of culture may all negatively affect hCEC propagation (Peh et al. 2013). Because of the latter, in most protocols, hCEC are isolated from several research-grade corneas and mixed at initiation of culture (Nakahara et al. 2013; Okumura et al. 2015), which may cause the results to be confounded by donor-to-donor variability. More importantly, this approach is not suitable for eventual clinical application of cultured hCEC because of lack of tissue traceability (Strong and Shinozaki 2010), and a possible higher risk of allograft rejection because the hCEC originated from different donors. Here, we show that with prior stabilization of DM–EC sheets from research-grade single donor corneas with extended storage time before culture (average 16 days) and a high donor age (average 69 years), we still were able to establish a successful culture in all cases, which was not possible for the non-stabilized contralateral corneas. This result is important for future clinical application of cultured hCEC. Because tissue traceability, and therefore, safety are maintained, the risk of rejection of the cultured hCEC is reduced and even with ‘unfavorable’ parameters, hCEC may be cultured successfully.

However, it should be pointed out that the study included only a small number of paired corneas (n = 7) of relatively old donors. Therefore it would be interesting to assess the effect of stabilization medium on growing hCEC in vitro from a larger number of donors, including donors with an age younger than 50 years since the latter have been shown to propagate better in culture than hCEC from older donors (Joyce 2003, 2012; Joyce and Zhu 2004). Furthermore, viability assays performed on freshly peeled DM–EC and during culture might enable quantification of viable and non-viable hCEC at any stage during the investigation and might confirm our hypothesis in more detail. Because a Trypan blue staining is not able to discriminate between apoptotic and dead cells (Perry et al. 1997), a thorough investigation of various staining methods is required in order to enable the qualitative assessment of the overall cell population prior to cell seeding.

In conclusion, we report a novel straightforward and practical manner to successfully culture hCEC derived from a single donor cornea. This procedure has obvious potential in improving in vitro hCEC culture protocols and may aid in the future clinical application of cultured hCEC for corneal endothelial diseases.
